# Infants Prefer Female Body Phenotypes; Infant Girls Prefer They Have an Hourglass Shape

**DOI:** 10.3389/fpsyg.2016.00804

**Published:** 2016-06-07

**Authors:** Gerianne M. Alexander, Laura B. Hawkins, Teresa Wilcox, Amy Hirshkowitz

**Affiliations:** Department of Psychology, Texas A&M University, College StationTX, USA

**Keywords:** eye-tracking, infants, body shape, toy preferences, waist-to-hip ratio

## Abstract

Adolescents and adults show preferences for male and female body shapes consistent with evolutionary theories of reproductive fitness and mate selection. However, when these preferences for females with narrow waists (i.e., 0.7 waist-to-hip ratio) and men with broad shoulders (i.e., mesomorphic body shape) emerge during the lifespan is largely unknown. To address this knowledge gap, eye-movements were tracked in 146 infants (3–18 months of age) during computer presentation of three-dimensional human figures varying in body features thought relevant for reproductive success (e.g., secondary sex characteristics, waist-to-hip ratio). When presented with pairs of figures differing in apparent sex, male and female infants looked significantly longer at the female figure compared to the male figure, a new finding that extends previous research showing preferences for female faces in infancy. When presented with same-sex figures differing in characteristics associated with mate value, male and female infants looked longer at a low mate value male (i.e., an endomorphic body type) compared to a high mate value male (i.e., a mesomorphic body type), a finding that replicates the results of previous research. In addition, the novel use of high and low mate value female figures showed a sex difference in visual attention, such that female infants looked longer at the high mate value female figure compared to the low mate female figure whereas male infants showed the opposite pattern of results. In sum, these findings suggest that infants generally do not possess preferences for adult-defined attractive male body shapes. However, infant girls’ greater attention to a female figure with an adult-preferred waist-to-hip ratio raises the possibility that evolved preferences for 0.7 waist-to-hip ratio influence girls’ later preference for toys representing females with an hourglass shape, perhaps supporting elaboration of adult social behaviors that enhance reproductive success (e.g., cooperative breeding).

## Introduction

Consistent with the predictions of evolutionary theory (e.g., Trivers, 1972, 1985), present day men generally prefer adult female sexual partners with traits associated with health and fertility, such as youth and physical attractiveness (Buss and Barnes, 1986; Kenrick et al., 1990; Townsend and Levy, 1990), whereas women generally prefer adult male sexual partners with traits associated with resource acquisition, such as financial success and social dominance (Sadalla et al., 1987; Kenrick et al., 1990; Townsend and Levy, 1990; Wiederman and Allgeier, 1992; Buss and Schmitt, 1993; Li et al., 2002). More recent research on the stability of sex differences in mate preferences indicate they are strongest for ideal partners (Eastwick et al., 2014) and sensitive to short- or long-term contexts (Little et al., 2014) and the fertility patterns of women (Gildersleeve et al., 2014).

The identification of high value mates in both sexes is informed by body characteristics that result from sexual differentiation. Prenatal hormones first determine the appearance of the genitalia (e.g., Siiteri and Wilson, 1973), a characteristic that signals the biological status of the individual as male or female. Later, increased production of gonadal steroids during pubertal development activates the expression of secondary sex characteristics (e.g., breast development in girls, facial hair in boys) that enhance recognition of biological sex and an adult reproductive status (Styne and Grumbach, 1978). In addition, hormone levels at puberty contribute to a pattern of body fat disposition that enhances human sexual dimorphism and signals reproductive fitness (Swami, 2006). Specifically, increased ovarian estrogens during female sexual maturation create an hourglass shape by facilitating fat deposition in the gluteofemoral region (thighs and buttocks) while inhibiting fat deposition in the abdominal region. In contrast, increased testicular androgens during male sexual maturation create an inverted triangle body shape that characterizes the muscular, mesomorphic male somatotype by facilitating fat deposition in the abdominal and upper body region while minimizing fat deposit in the gluteofemoral region (Björntorp, 1987; Leibel et al., 1989).

Individual differences in male and female body fat composition are commonly quantified by the ratio of the circumferences of the waist and hips (WHR) (Singh, 1993). Larger WHRs in both sexes are associated with poorer health outcomes, including increased risk for cardiovascular disorders and other systemic disease (Guagnano et al., 2001; De Koning et al., 2007). Women’s WHRs also convey information about fertility, such that low WHRs (0.7) predict earlier, regular menstrual cycles (Van Hooff et al., 2000; Lassek and Gaulin, 2007) and higher rates of conception (for review, see Singh et al., 2010). Appraisals of potential mates are influenced by WHRs: men typically prefer women with a 0.7 WHR (i.e., the hourglass shape) and women typically prefer men with a WHR of 0.9 consistent with the muscular, mesomorphic body shape (e.g., Horvath, 1979; Franzoi and Herzog, 1987; Henss, 1995; Singh, 1995; Furnham et al., 1997; Lynch and Zellner, 1999; Dixson et al., 2003; Saad, 2008; Puts, 2010).

Although most research on WHRs focuses on their role in adult mate selection (e.g., Singh, 1993), three possible ontogenetic models are proposed to account for the proximate causes of their development (Connolly et al., 2004; Saxton et al., 2006). One hypothesis is that adult-typical WHR preferences exist from birth, suggesting that sexual differentiation in prenatal or perinatal life is sufficient to explain their development. A second hypothesis is that sex-linked WHR preferences emerge gradually across childhood, consistent with a role of postnatal experiential factors. Finally, a third hypothesis is that WHR preferences emerge coincident with the pubertal onset of sex differences in fat disposition, suggesting that the known influences of increased gonadal hormones on cognitive-emotional aspects of sexual motivation (e.g., sexual desire or interest) (Alexander and Sherwin, 1993; Alexander et al., 1994; Lee et al., 2003) may extend to preferences for physical characteristics signaling high mate value.

Empirical tests of these three hypotheses are limited. An investigation of infant preferences for related male somatotypes (Heron-Delaney et al., 2013) found no somatotype preferences until 9 months of age. At that age, infants preferred an endomorphic (i.e., fatter) male figure over a mesomorphic (i.e., muscular) male figure, a finding attributed to children’s greater familiarity with overweight adults in our present-day society. In a second study, children’s self-reported preferences for WHRs of 0.7, 0.8, and 0.9 were measured using line drawings of adult human figures (Connolly et al., 2004). At 6 and 8 years of age, both sexes reported similar WHR preferences for male and female figures, with average scores ranging between 0.8 and 0.9 (i.e., more male-typical). Adult-typical preferences for the male WHR of 0.9 emerged first at 10 years of age, whereas adult-typical preferences for the female WHR of 0.7 emerged at 12 years of age. As adult-typical WHR preferences in this research were not evident in the youngest children, the results provide no support for the first two hypotheses. Rather, the emergence of self-reported WHR preferences around the typical ages of adrenarche and puberty is consistent with the proposal that WHR preferences are dependent on sexual maturation (Connolly et al., 2004).

In sum, the findings of two previous investigations of early mate-relevant body preferences generally support the third hypothesis; that adult-typical preferences for WHRs in male and female body shapes emerge coincident with sexual maturation. Although findings from the infant study suggest experiential factors may contribute to human body shape preferences, these developing preferences are contrary to those that emerge in later life. However, as no studies of infants have examined preferences for male vs. female phenotypes or measured relative preferences for female figures varying in WHRs, it may be premature to conclude that all adult-typical preferences emerge with sexual maturation. To address this knowledge gap, the current study used eye-tracking technology to measure infant attention to human body shapes varying in phenotypic sex and mate value, as indicated by WHR. As greater familiarity with female caregivers has been proposed to explain preferences for other sexually dimorphic body features, such as female faces (Quinn et al., 2002), we hypothesized infants presented with adult male and adult female body shapes would attend more to the adult female phenotype. Based on the results of previous research on within-sex body shape preferences, we also hypothesized that an infant preference for male body shapes with higher relative fatness (i.e., lower mate value, larger WHR) would generalize to the female body shape with higher relative fatness (i.e., lower mate value, larger WHR).

## Materials and Methods

### Participants

Healthy and full-term infants (*n* = 146) recruited primarily from commercially produced lists completed a protocol approved by the university Institutional Review Board. All parents provided informed consent in accordance with the Declaration of Helsinki. Young infants (3–7 months of age) included 20 males (Mean = 4.68, *SD* = 0.78) and 27 females (*M* = 4.96, *SD* = 0.95). Intermediate infants (8–13 months of age) included 19 males (*M* = 11.75, *SD* = 0.73) and 31 females (*M* = 11.74, *SD* = 1.02). Older infants (14–18 months of age) included 17 males (*M* = 15.32, *SD* = 1.23) and 32 females (*M* = 15.36, *SD* = 1.45). Parents reported their infants’ race/ethnicity as Caucasian (79.6%), Hispanic (14.3%), Black (2.0%), or of mixed race/other (4.1%). Seventeen additional infants were tested but excluded from the sample due to fussiness or procedural problems. Following completion of the study, parents received $5 or a lab T-shirt for participation.

### Apparatus and Data Recording

Infants sat on their parent’s lap approximately 65 cm away from a remote eye tracker (Tobii T60 XL). The infrared corneal reflection eye tracker was embedded in the lower portion of a 24 in flat screen monitor (17.7W TFT l flat screen monitor) (resolution: 1024 × 768 pixels) and detected the position of the pupil and the corneal reflection of the infrared light from both eyes. The Tobii T60 XL records data at 60 Hz with an average accuracy of 0.5° visual angle and a head movement compensation drift of G0.1. The monitor was mounted on an adjustable arm so that it could be positioned optimally for each infant.

Fixation data were defined using the Tobii fixation filter (version 2.2.8) with a velocity threshold of 35 pixels and a distance threshold of 35 pixels. Total duration of looking was calculated by the sum of fixation data for each trial. Prior to test, each infant was calibrated by registering five gaze positions covering over 80% of the viewing area. After calibration, infants immediately viewed the test displays. The test displays were presented using professional visualization software (Tobii Studio) on a Dell Precision T5500 desktop computer with a Windows 7 operating system.

### Stimuli and Design

The stimuli were human figures adapted from a previous investigation of mate selection in adults (Maner et al., 2008) and made three-dimensional for research on the motivational value of human figures (Charles et al., 2013) using Autodesk Maya 3D and Adobe After Effects. Each human figure was 6 cm × 13.5 cm, dressed in identical t-shirts and pants, and shown on a light blue background measuring 15.3 cm by 25.5 cm.

**FIGURE 1:**
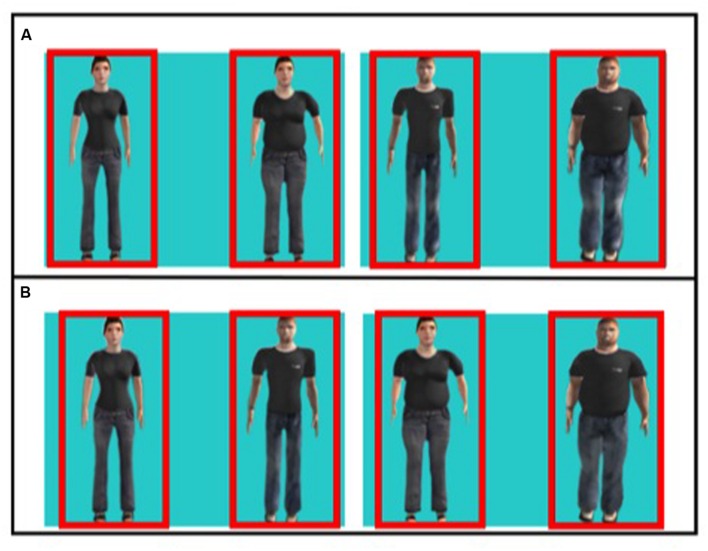
**Examples of the two sets of test stimuli.** **(A)** Males vs. female phenotypes matched for mate value. *Left pair:* two female figures, small WHR and large WHR respectively. *Right pair:* two male figures, mesomorph and endomorph, respectively. **(B)** High vs. low mate value body types matched for sex. *Left pair:* high mate value, female and male respectively. *Right pair:* low mate value, female and male respectively.

Infants viewed two sets of displays depicting pairs of human figures. One set paired an adult male and adult female phenotype matched for mate value conditions; namely a endormophic male paired with a high WHR female (low mate value condition) and a mesomorphic male paired with a low WHR female (high mate value condition). A second set paired high and low mate value phenotypes matched for sex. The male condition paired a mesomorph (i.e., high mate value) with an endomorph (i.e., low mate value). The female condition paired a 0.7 WHR (i.e., high mate value) with a larger WHR (i.e., low mate value). The presentation side (right or left) of figures in a pair was counterbalanced across trials and order of trials was counterbalanced across infants. Each display was presented for 4 s, for a total of 32 s. **Figure 1** shows examples of the two sets of stimuli.

### Data Coding

Each figure was defined as an area of interest (AOIs). Consequently, each slide had two AOIs of equal size, one on the left and one on the right. Number of fixations and duration of looking to the AOIs were both coded. Both metrics yielded similar results, and so only duration of looking is reported. Mean substitution for missing data (i.e., less than 2% of the data) was applied to maximize the sample size.

## Results

### Male vs. Female Phenotypes Matched for Mate Value

**FIGURE 2:**
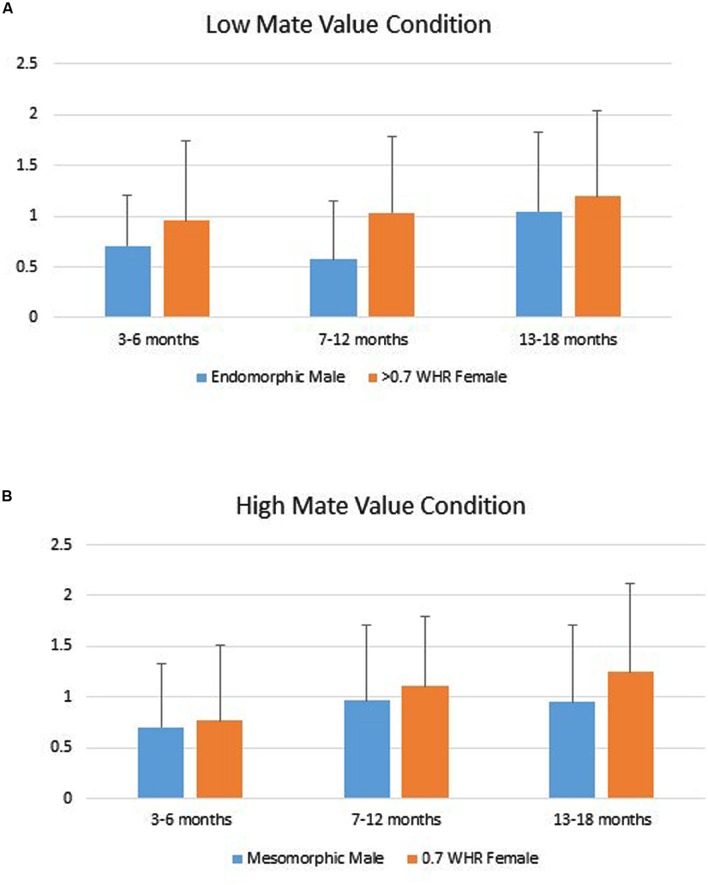
**(A)** Infants’ mean (SD) duration of looking to the low mate value male (endomorph) and female (>0.7 WHR) figures. **(B)** Infants’ mean (SD) duration of looking to the high mate value male (mesomorph) and female (0.7 WHR) figures.

Infants’ duration of looking to the AOIs (DV) was analyzed using a repeated measures ANOVA with Phenotype (male vs. female) as a repeated factor, Age Group (young, intermediate, older) and Gender (boy vs. girl) as grouping factors within levels of Mate Value (high vs. low). That analyses showed only a significant main effect for Phenotype, Multivariate *F*_2,139_ = 4.82, *p* = 0.009. Tests of within-subjects effects showed the Phenotype effect was significant for both the high, *F*(1,140) = 5.34, *p* = 0.022, Cohen’s *d* = 0.22 and low Mate Value conditions, *F*(1,140) = 4.10, *p* = 0.045, *d* = 0.19. In both, infants looked longer to the female figure compared to the male figure (**Figure 2**).

### High vs. Low Mate Value Body Types Matched for Sex

**FIGURE 3:**
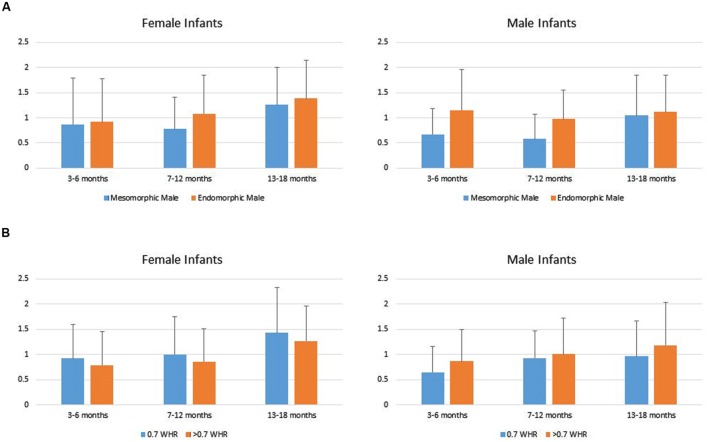
**(A)** Female and male infants’ mean (SD) duration of looking to the male high (mesomorphic) and low (endomorphic) mate value figures. **(B)** Male and female infants’ mean (SD) duration of looking to the female high (0.7 WHR) and low (>0.7 WHR) mate value figures.

Data from two infants in the youngest age group were lost due to fussiness, reducing the total sample size to 144. Infants’ duration of looking to the AOIs (DV) was analyzed using a repeated measures MANOVA with Mate Value (high vs. low) as a repeated factor, Age Group (young, intermediate, older) and Gender (boy vs. girl) as grouping factors within levels of Phenotypic Sex (male vs. female). That analyses showed a significant main effect for Age Group, Multivariate *F*_4,276_ = 3.83, *p* = 0.005, and a Mate Value × Gender interaction, Multivariate *F*_2,137_ = 3.50, *p* = 0.033. Univariate tests showed the main effect of Age Group occurred because looking times generally increased across the three groups of infants (Young infants: *M* = 0.823, *SD* = 0.64; Intermediate infants: *M* = 0.915, *SD* = 0.67; Older infants: *M* = 1.24, *SD* = 0.75). The tests of within subject effects for the male condition showed no significant Mate Value × Gender interaction *F*(1,142) = 1.21, *p* = 0.27, and a main effect of Mate Value *F*(1,138) = 9.59, *p* = 0.002, such that both male and female infants looked significantly longer to the endomorph than to the mesomorph, *d* = 0.31. The tests of within subject effects for the female condition showed only a significant Mate Value × Gender interaction, *F*(1,142) = 5.573, *p* < 0.05. Follow-up *t*-tests showed no significant difference in male and female looking times to the large WHR female, *t*(142) = -0.218, ns, *d* = 0.03. However, female infants compared to male infants looked longer to the 0.7 WHR female, *t*(142) = 2.26, *p* = 0.025, *d* = 0.41 (**Figure 3**).

## Discussion

Eye-tracking measures of infant looking times in this research showed an early emergence of between- and within-sex human body shape preferences. Consistent with an established infant preference for female faces (Quinn et al., 2002), when presented with pairs of male-typical and female-typical adult human figures, boys and girls ranging in age from 3 to 18 months looked longer at the female phenotype. When presented with pairs of same-sex human figures differing in physical characteristics known to influence adult assessments of mate value, boys and girls looked longer at the adult male phenotype with a lower mate value. Boys also looked longer at the female phenotype with a lower mate value, consistent with the suggestion that infants show greater visual interest in adult body shapes with higher fat content. Unexpectedly, although girls shared boys’ preference for a lower mate value male figure, they looked longer at a higher mate value female figure defined by a smaller, adult preferred WHR.

Previous research indicates infants are sensitive to other sex-linked perceptual cues, such as voice (Miller et al., 1982), well before the age when they can reliably categorize human bodies as male or female (i.e., 2 years of age) (Johnston et al., 2001). Although some research suggests infants do not acquire sensitivity to body structure until the end of the first year of life (Slaughter et al., 2002), findings from ERP research are consistent with the recognition of normal body configuration at 3 months of age (Gliga and Dehaene-Lambertz, 2005). Additionally, other research using three-dimensional representations of human figures found infants at 3.5 months of age are sensitive to the arrangement and size of female body parts (i.e., the relative length of the legs and waists) (Zeiber et al., 2015). Our novel finding that infants prefer an adult female phenotype relative to an adult male phenotype is consistent with this early sensitivity to the size and shape of human body features. Moreover, we found infants across the three age groups showed this preference for females in both high and low mate value conditions, a pattern of response indicating a sensitivity to body features signaling biological sex in human figures with both high and low sexual dimorphism, respectively. Of course, whether an infant preference for the female phenotype observed in this research would extend to prepubertal body shapes with even lower sexual dimorphism is a question for future research.

The primary goal of this research was to inform theories of the ontogeny of adult-typical preferences for high mate value body characteristics by examining eye-movements in infants during presentation of three-dimensional renderings of human figures. With the exception of the sex difference observed in infant response to the female figures differing in WHRs, our data are consistent with earlier evidence indicating sexual maturation is required to trigger self-reports of adult-typical body shape preferences (Connolly et al., 2004). Stronger tests of the hormonal hypothesis would include future research examining whether self-reports of adult-typical body preferences occur at younger ages in children with precocious puberty and at later ages in adolescents with delayed pubertal development, for example as a consequence of endocrine disorders (e.g., Kenneth et al., 1977) or extreme physical training (Warren, 1979; Lindhom et al., 2011). Whereas biological factors are proposed to account for WHR preferences in older individuals, the proposed determinants of infant body preferences are largely experiential. Infant preferences for female over male faces, for example, are generally understood as the effect of primary caretaker gender on the early representation of human faces (Quinn et al., 2002) and it seems reasonable to propose that a similar mechanism may also contribute to the observed preference for the female-typical body shape. Increased exposure to overweight adults in contemporary society, another experiential factor, is hypothesized to explain infant preferences for male adult body shapes with a higher fat content (Heron-Delaney et al., 2013), a possibility that would also explain our finding that boys looked longer at both male and female figures representing low mate value. However, unless future research establishes that boys and girls differ in their experience with overweight women but not overweight men or that girls but not boys are responsive to contemporary cultural standards of female beauty beginning at 3 months of age, other factors are required to explain girls’ preference for an adult female figure with the smaller, adult-preferred WHR.

There is no existing theoretical narrative that can explain the sex difference we observed in attention to female figures. However, the apparent consistency between infant girls’ interest in the high mate value female with an hourglass shape and older girls’ preference for toys representing women with an hourglass shape (i.e., Barbie) (Karniol et al., 2012) suggests an understanding of our results can be informed by research on gender-linked toy preferences. In other research measuring visual attention, female infants compared to male infants look longer at a doll than a toy truck (Alexander et al., 2009; Lauer et al., 2015). Later, boys prefer interacting with toy vehicles and girls prefer interacting with dolls (for discussion, see Alexander and Saenz, 2012). Although gender-linked toys (e.g., vehicles, baby dolls) are clearly cultural artifacts associated with contemporary social roles for men and women (Liben and Bigler, 2002), non-human primates also show similar sex differences in response to these gender-linked objects (Alexander and Hines, 2002; Hassett et al., 2008). One general understanding of these common primate interests is that they represent evolved preferences that support the development of behaviors with adaptive significance. For example, an early and sustained female interest in toy replicas of babies may encourage abilities that support later nurturance and offspring survival (e.g., Alexander and Hines, 2002). From this theoretical perspective, our finding of greater visual attention to a high mate value female phenotype in female infants – coupled with girls’ later strong preference for adult female dolls with an hourglass shape and their self-reports of this preference around the time of sexual maturation (Connolly et al., 2004) – is converging evidence of an evolved preference for a female body feature signaling health and fertility, one that may also support later adaptive behavior. The functional significance of this preference in girls will clearly require further research on the influence of early WHR preferences on later reproductive behavior. An attentional bias toward relevant others, such as the pregnant or lactating female, is proposed to support the development of social competencies required for cooperative breeding success (Burkart and van Schaik, 2010). One possibility, then, is that a female interest in female figures signaling fertility may represent a related attentional bias, one that allows the encoding of behaviors supporting successful pregnancy and provides a foundation for cooperative breeding success.

## Author Contributions

GA and TW conceptualized the research. All authors contributed to the design, acquisition, and analyses of the research. GA, LH, and TW were responsible for the interpretation of the data and preparation of the manuscript.

## Conflict of Interest Statement

The authors declare that the research was conducted in the absence of any commercial or financial relationships that could be construed as a potential conflict of interest.
